# Deducing corticotropin-releasing hormone receptor type 1 signaling networks from gene expression data by usage of genetic algorithms and graphical Gaussian models

**DOI:** 10.1186/1752-0509-4-159

**Published:** 2010-11-19

**Authors:** Dietrich Trümbach, Cornelia Graf, Benno Pütz, Claudia Kühne, Marcus Panhuysen, Peter Weber, Florian Holsboer, Wolfgang Wurst, Gerhard Welzl, Jan M Deussing

**Affiliations:** 1 Max Planck Institute of Psychiatry, Kraepelinstr. 2-10, 80804 Munich, Germany; 2 Helmholtz Centre Munich, German Research Centre for Environmental Health, (GmbH) and Technical University Munich, Institute of Developmental Genetics, Deutsches Zentrum für Neurodegenerative Erkrankungen (DZNE), Ingolstädter, Landstraße 1, 85764 Munich-Neuherberg, Germany

## Abstract

**Background:**

Dysregulation of the hypothalamic-pituitary-adrenal (HPA) axis is a hallmark of complex and multifactorial psychiatric diseases such as anxiety and mood disorders. About 50-60% of patients with major depression show HPA axis dysfunction, i.e. hyperactivity and impaired negative feedback regulation. The neuropeptide corticotropin-releasing hormone (CRH) and its receptor type 1 (CRHR1) are key regulators of this neuroendocrine stress axis. Therefore, we analyzed CRH/CRHR1-dependent gene expression data obtained from the pituitary corticotrope cell line AtT-20, a well-established *in vitro *model for CRHR1-mediated signal transduction. To extract significantly regulated genes from a genome-wide microarray data set and to deduce underlying CRHR1-dependent signaling networks, we combined supervised and unsupervised algorithms.

**Results:**

We present an efficient variable selection strategy by consecutively applying univariate as well as multivariate methods followed by graphical models. First, feature preselection was used to exclude genes not differentially regulated over time from the dataset. For multivariate variable selection a maximum likelihood (MLHD) discriminant function within GALGO, an R package based on a genetic algorithm (GA), was chosen. The topmost genes representing major nodes in the expression network were ranked to find highly separating candidate genes. By using groups of five genes (chromosome size) in the discriminant function and repeating the genetic algorithm separately four times we found eleven genes occurring at least in three of the top ranked result lists of the four repetitions. In addition, we compared the results of GA/MLHD with the alternative optimization algorithms greedy selection and simulated annealing as well as with the state-of-the-art method random forest. In every case we obtained a clear overlap of the selected genes independently confirming the results of MLHD in combination with a genetic algorithm.

With two unsupervised algorithms, principal component analysis and graphical Gaussian models, putative interactions of the candidate genes were determined and reconstructed by literature mining. Differential regulation of six candidate genes was validated by qRT-PCR.

**Conclusions:**

The combination of supervised and unsupervised algorithms in this study allowed extracting a small subset of meaningful candidate genes from the genome-wide expression data set. Thereby, variable selection using different optimization algorithms based on linear classifiers as well as the nonlinear random forest method resulted in congruent candidate genes. The calculated interacting network connecting these new target genes was bioinformatically mapped to known CRHR1-dependent signaling pathways. Additionally, the differential expression of the identified target genes was confirmed experimentally.

## Background

The neuropeptide corticotropin-releasing hormone (CRH), discovered in 1981, is the key regulator of the hypothalamic-pituitary-adrenal (HPA) axis [1] and orchestrates the neuroendocrine, autonomic and behavioral responses to stress [2]. Stress and disturbances in the CRH system, i.e. hyperactivity and impaired negative feedback regulation of the HPA axis, are frequently accompanying psychiatric disorders including depression and anxiety [3-5]. The CRH system has been extensively studied applying genetically engineered gain-and loss-of-function mouse models underscoring its importance for the development of psychiatric disorders [6-8].

The two CRH receptors, CRHR1 and CRHR2, are class B G protein-coupled seven transmembrane receptors that are capable of activating different G proteins and signaling cascades upon ligand-binding. The dominant CRHR1-activated signaling pathway in endogenous and recombinant cell lines is the adenylyl cyclase-protein kinase A (PKA) pathway via Gα_s _[9,10]. Dependent on species, tissue and cell type, both receptors are known to activate Gα_q_/phospholipase C (PLC)-, AKT/PI3 kinase-, NOS/guanylyl cyclase-, caspase pro apoptotic-and NFKB or NURR1/NUR77 transcription factor signaling pathways [11]. In AtT-20 cells, a mouse corticotrope pituitary tumor cell line expressing CRHR1, PKA activation on the one hand triggers Ca^2+^-dependent signaling via CamKII, which increases NUR77 and NURR1 transcription [12]. On the other hand, PKA activates a mitogen-activated protein kinase (MAPK) pathway including RAP1, B-RAF, MEK1 and extracellular signal-regulated kinase (ERK) 1/2 resulting in NUR77 phosphorylation/transactivation and transcription of proopiomelanocortin (POMC). In specific brain areas such as the hippocampus CRH activates ERK1/2 via CRHR1, whereas in hypothalamic nuclei and the central nucleus of the amygdala CRH triggers other signaling pathways as no CRH-dependent ERK1/2 phosphorylation was detected [13]. CRHR1, as key regulator of the neuroendocrine and behavioral responses to stress, has attracted major interest as a potential novel target for the therapeutic intervention in major depressive disorder [14-17]. However, CRH/CRHR1-dependent signal transduction mechanisms are only partially understood. Therefore, a more precise understanding of the involved intracellular signaling mechanisms is a prerequisite towards the development of efficient and less pleiotropic CRHR1-specific antagonists [18].

The activation of specific signaling pathways will cause changes in gene expression signatures. Changes at transcriptional level normally precede changes at protein level and provide an entry point to understand the underlying regulatory networks. Expression profiling applying high-throughput microarray technology allows monitoring thousands of genes simultaneously and to characterize changes in gene expression patterns induced by a defined stimulus on a genome-wide scale. In order to dissect signaling mechanisms of the CRHR1 in depth we used AtT-20 cells, which are a well established *in vitro *system to study CRHR1 signaling [12,19,20]. To gain insight into the dynamics of CRH-/CRHR1-dependent signaling pathways we investigated the alterations in expression patterns after CRH treatment at five different time points between 1 and 24 h on the Max Planck Institute of Psychiatry (MPIP) 24 k cDNA microarray platform [21].

For the analysis of expression profiling data a plethora of methods has been developed in order to rank genes by t-statistics [22-24]. Applying these univariate feature selection methods the most significantly regulated genes can be determined, but variables (genes) are always considered in isolation. Our aim was to predict gene-gene interactions between candidate genes that are significantly regulated within the time course by sequentially using univariate as well as multivariate variable selection methods and afterwards graphical models. Multivariate variable selection was considered of importance because variables (genes) contribute only in combination with other variables to the discrimination of the input data rather than in isolation. For variable selection we used a maximum likelihood discriminant method (MLHD), which is equivalent to linear discriminant analysis (LDA) combined with a genetic algorithm (GA) [25]. The method combines a small number of five variables (genes) into subsets (chromosomes) mimicking biological crossover and mutation for computation of the discriminant function. Due to computational limitations to determine all possible chromosomes out of the complete set of variables a stochastic search strategy for feature selection is necessary. GA procedures in combination with classification methods have been successfully used in the analysis of microarray data [26,27]. Other optimization procedures implementing classification methods such as the greedy algorithm and simulated annealing were also investigated here and in the past [28-30]. To oppose MLHD embedded in GA to selection procedures which are in principle not affected by the dimensionally problem (small sample size compared to large variable number) also random forest was used. Subsequently, graphical Gaussian models (GGMs) have been applied to a small subset of genes in order to derive genetic interactions [31]. The resulting putative gene-gene interactions from the graphical model were assigned to signaling pathways activated by CRH/CRHR1 via text mining methods.

## Results and Discussion

### Identification of candidate genes

AtT-20 cells are a widely used and best studied *in vitro *model to investigate CRHR1-dependent signal transduction. As pituitary-derived corticotrope cell line, AtT-20 cells express CRHR1 but not CRHR2 [20] which permits specific analyses of CRHR1 signaling. A plethora of molecules regulated downstream of CRHR1 have been identified and studied in this cell line [12,19,20], however, the complex system of CRHR1-regulated signaling cascades is not fully understood. To further elucidate genes involved in CRH-activated signaling pathways we treated the cells with 100 nM CRH at five different time points (1, 3, 6, 12, 24 h). The dose of 100 nM CRH was chosen as 100 nM CRH evokes a response in AtT-20 cells but is still below the concentration of maximal stimulation observed in transactivation assays [20]. Within the first 3 h CRH is known to activate immediate early genes such as c-Fos [20]. With the first two time points this immediate early effect of CRH was investigated whereas at 6 and 12 h the late CRH response was analysed. Furthermore, we were interested in the long-term effects of 24 h of continuous CRH treatment.

The gene expression data obtained by microarray analysis of CRH-treated vs untreated AtT-20 cells at different time points have been deposited in NCBI's Gene Expression Omnibus (GEO) [32] and are accessible by the GEO Series accession numbers GSE13156 and GPL7467.

**Figure 1:**
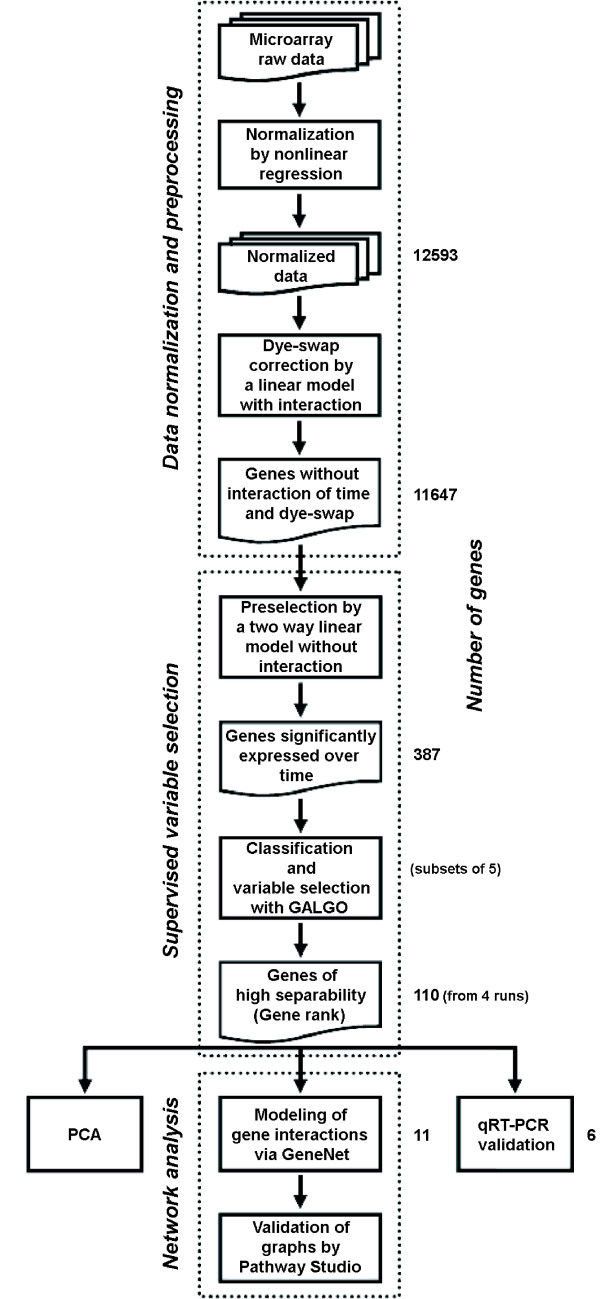
**Workflow scheme demonstrating stepwise analysis of the microarray data**.

Candidate genes were selected following (i) data normalization and preprocessing, (ii) a preselection process and (iii) supervised variable selection (Figure 1). By statistical tests it has been verified that prerequisites for the two-way ANOVA like normal distributed expression ratios and equal variances across samples are fulfilled. Furthermore, a balanced design was chosen in the present microarray study meaning equal group size of six technical replicates for each time point. As the technical replicates were performed on six different arrays, the data sets are independent.

### Normalization and Dye-swap correction

MA-plots of the spot signals from 48 pins (encoded by different colors) before and after the normalization procedure are shown in Additional File 1 (exemplified by time point 12 h after CRH treatment). The normalization procedure was successful as the loess fit curves in the MA-plots of the transformed array data (i.e. the difference between measured and predicted M-ratios) show nearly horizontal lines meaning that most of the genes do not show much change in their intensity ratios as expected [33]. After normalization, signals of 12593 spots measured at each time point were tested applying a linear model to exclude genes showing significant dye-dependent effects in their expression profile over time. A microarray design based on fixed effects parameters which was applied here was originally described by Kerr and Churchill [34]. According to this fixed effects model differential expression values of 946 genes (p < 0.01 and false discovery rate FDR < 0.13) with a significant interaction of the factors time and dye-swap were excluded from the dataset.

### Preselection 2-way ANOVA

It is important to further restrict microarray data before multivariate analysis since most of the genes do not show dynamic differential expression over time following CRH stimulation and thus do not contribute to the discrimination between classes. By applying a two-way linear model without interaction of the factors time and dye-swap, genes were preselected for supervised clustering and variable selection with GA/MLHD. Because one of the main assumptions of the ANOVA is equal variances across groups we applied Levene's test for homogeneity of variance. For the predominant part of the gene expression ratios it has been verified by Levene's test that there is no shift in variation resulting in 450 of 11647 tests with p < 0.1% and a maximum FDR of 2.5%. By the two-way ANOVA utilized in the preselection process, 387 genes (p < 0.01 and false discovery rate FDR < 0.3) were identified as significantly regulated over time. This reduction of the feature space was proposed by many authors [35,36] to improve the predictive power of the classifier. In principle, due to the small number of genes in a subset (chromosome) considered as training data for classification, a reduction of the initial data set is not necessary. However, stochastic searches such as genetic algorithms are able to detect only a small part of the total solution space. To reduce the solution space and to generate more stable results, we used the preselected set of 387 genes as input for the GALGO program. Before, Shapiro-Wilk's tests (also applied by Karlovich et al. [37]) were performed to demonstrate that the expression ratios were log_2_-normally distributed for the preselected genes as well as for the genes used as input for the ANOVA. Considering the corresponding preselected gene expression ratios 3 of 387 tests showed p < 0.1% with a maximum FDR of 10% while regarding the whole data set used in the two-way ANOVA for feature preselection 175 of 11647 tests resulted in p < 0.1% and a maximum FDR of 6.6%.

### GA/MLHD

The general application of the maximum likelihood (MLHD) classifier implemented in a genetic algorithm to microarray datasets was demonstrated by Trevino and Ooi [25,26]. In addition it was shown that for a chromosome size of five variables as used in the present study the classification error resulting from MLHD is similar compared to other classifiers (e.g. KNN, SVM, NC) (see Table three (Appendix) of [25]).

Additional File 2 shows the complete list of 110 transcripts derived from four independent GALGO analyses based on the preselected genes including all genes occurring at least once within the top 50 ranks. The frequency rank of each gene was determined by counting the chromosomes with the respective gene reaching a classification accuracy of 100% (goal fitness). In total, there are 15 genes that occur in all four runs among the topmost 50 ranked genes. Excluding those genes that were not fully annotated, a total of 10 genes remained in four runs among the topmost ranked genes. One exception is the addition of Hmgcs1 because it was detected twice (Spot ID 6705 and 16977) in at least three GALGO analysis results. Hence, 11 unique candidate genes, which contributed strongly to the discrimination between groups (time points) were selected for further validation by qRT-PCR.

With multivariate selection procedures variables (i.e. gene expression ratios) are tested in combination to identify interactions between them. Detailed inspection of the chromosomes (subsets of 5 genes) revealed that often combinations of candidate genes with complete discrimination occurred in the GALGO analysis results (e.g. 4 candidates out of 11 in 224 unique chromosomes of analysis 1). Therefore, the selected genes are expected to be highly correlated with each other in terms of gene interaction networks. The dependency of top ranked GA (GALGO) selected feature components with each other (i.e. interaction networks) was also investigated by metabolic profiling studies with help of mass spectrometry [38]. The authors conclude that preliminary hypothesis can be generated based on GA selected features (genes) however it is also important to consider more complete knowledge of biological pathways from e.g. public databases or text mining tools.

### Reliability of candidate genes

Feature selection is critical when LDA is applied to microarray datasets where the number of genes (p) is distinctly larger than the number of samples (n) because overfitting can easily occur. To solve this problem we investigated several optimization algorithms for feature selection based on MLHD or LDA considering subsets of variables in which the number of genes is smaller than the sample size (p < n). The minimum sample size suggested is five observations per independent variable ([39] p. 258). Each chromosome of the GA/MLHD and SANN/LDA approach has a 6:1 ratio of observations to predictor variables, which meets the 5:1 ratio recommended. Furthermore, generalization curves based on the preselected expression ratios showed that a chromosome size of five is far away from a situation of overfitting because for a number of variables greater than 20 the test error (red curve) increases while the training error (black curve) do not change much (Additional file 3).

We also took random forest classification into account, which is suited for datasets where the number of variables is larger than the number of samples (p > > n).

### Greedy/LDA

We performed the greedy feature selection and applied LDA to the selected gene expression ratios. Each leave-one-out training set consisted of 5 to 10 genes and was achieved by repeating the F-test as long as the difference between two statistical models was significantly low. The LOOCV classification error was 23%, which is in the same range as shown by Wang et al. [30] using greedy-LDA selection methods. A ranked list was generated based on the frequency of each gene in the training sets of the samples. The selected genes were compared to the extracted features from the GA/MLHD procedure, and an overlap of 59% (10/17) was determined (Table 1). In case of the 11 candidates 7 genes of the greedy feature selection procedure coincide and in case of the top 6 validated candidates 5 genes overlapped, only Acsl4 is missing. In summary, both feature selection methods resulted in a clear overlap of the selected genes. The advantage of a greedy algorithm is the requirement of much less computational resources and that it is faster to execute. On the other hand, the greedy algorithm does not reach always a global optimal solution [40]. To address this limitation we tested also simulated annealing, another optimization search algorithm, for the validation of the GA/MLHD results.

**Table 1:** Comparison of RF, Greedy/LDA and SANN/LDA with GA/MLHD (from GALGO)

	Gene set	Overlap with GA/MLHD^1)^	Overlap with 11 candidates	Overlap with the top 6 validated candidates
RF	387	12/17	8/11	5/6^2)^
Greedy/LDA	5 -10	10/17	7/11	5/6^3)^
SANN/LDA	5	17/17	11/11	6/6

^1) ^Based on 17 genes from 4 GALGO runs among the top 50

^2) ^Pex13 missing

^3) ^Acsl4 missing

### SANN/LDA

In contrast to a greedy algorithm which often leads to a local optimum, simulated annealing (SA) derived from statistical mechanics converges to global optimum solution. In this SA approach a fitness function (generalized energy) was applied evaluating the classification error by using LOOCV and LDA instead of the physical energy. SA operates as a probabilistic hill-climbing procedure searching for the global optimum of the fitness (target) function. Simulated annealing has previously been demonstrated to be suitable for classification of gene expression data from microarrays by training of an artificial neural network [41]. To select gene expression ratios for the calculation of fitness values in the optimization process and to constrain the search space we developed an algorithm for input selection of subsets of variables (INSEL). A similar approach with the purpose of aggregating an ideally minimal subset of inputs with strong discriminative capability was described by Filippone et al. [28,29]. Details about our R-code for input selection can be found in the Additional File 4. Altogether 65 transcripts resulted from four separate SANN/LDA analyses including all genes occurring at least once within the top 50 genes ranked by their frequency in chromosomes, which reached a classification accuracy of 100%. 52 out of these 65 transcripts were in accordance with the 110 transcripts from four GALGO runs representing an overlap of 80%. Further comparison of the results of both feature selection methods SANN/LDA and GA/MLHD revealed identical selected genes (17/17) including all candidates (11/11) as well as the validated candidate genes (6/6) (Table 1). With SANN/LDA we reached a better overlap than in case of greedy/LDA and GA/MLHD. We ascribe this higher accordance of selected features by SANN/LDA and GA/MLHD to a more extensive variable combination caused by a similar evolutionary algorithm of both methods compared to greedy/LDA. For SANN/LDA we chose the same input parameters i.e. chromosome size, amount of solution chromosomes and fitness score just as for GA/MLHD. One of the main differences of simulated annealing compared to genetic algorithm is the use of only mutations in chromosomes whereas the genetic algorithm in addition takes the combination of two parent chromosomes (crossover) into account. In summary, despite of the more sophisticated search procedure in the genetic algorithm we obtained a high overlap between the respective top 50 selected genes of SANN/LDA as well as GA/MLHD and therefore validated the GALGO results. A further SANN/LDA analysis using a chromosome size of three instead of five revealed also a high agreement of selected genes (15/17, LOOCV < 15%) with GA/MLHD confirming the stability of the results (data not shown).

Similar to the discriminant vector in classical LDA a supported vector machine (SVM)-based approach performs gene selection using a weight vector. One approach for gene selection using SVM is the Recursive Feature Elimination (RFE) introduced by Guyon et al. [42]. Filippone et al. implemented the classification method SVM in their input selection algorithm [28,29]. We also tested SVM in our feature selection algorithm but the SANN/SVM method showed distinctly more chromosomes used for classification with a higher LOOCV error rate than SANN/LDA (data not shown). Another advantage of LDA compared to SVM implemented in the SA based gene selection method (INSEL) is that it requires much less computational resources and it is faster.

### Random forest

To contrast the outcome of the GA/MLHD feature selection procedure with classification methods proposed to be not affected by the dimensional problem (p > > n), which is the case for microarray data analysis we applied also tree classifiers. Random forest (RF) represents an algorithm for classification which uses an ensemble of classification trees [43]. Each classification tree is generated by selecting a bootstrap sample of the data, and at each split predictor variables are randomly selected. Therefore, random forest includes bagging [43,44], i.e. bootstrap aggregating, as well as random variable selection for tree building. Gene selection and classification of microarray data via RF has been successfully applied by Díaz-Uriarte and Alvarez de Andrés [45]. In RF feature selection procedures both the permutation and the Gini importance can be used to determine the relevance of each variable [46]. The mean decrease in Gini criterion was computed for each variable (gene) obtained by RF analysis using the 387 expression ratios of the preselected genes. The resulting list was sorted according to the Gini index in descending order and the top most 50 genes were compared to the selected genes by GA/MLHD. A considerable overlap of the top ranked genes of the RF analysis with the selected genes by GALGO of up to 71% (12/17) was determined (Table 1) and a similar overlap of up to 65% (11/17) by utilization of the permutation importance (data not shown). Furthermore, we found 8 of the 11 candidate and 5 of the 6 validated candidate genes (Pex13 is missing) by the Gini importance ranked lists with an out of bag (OOB) classification error of 13.3%. We conclude from the good agreement of the selected genes in both feature selection methods that classical LDA or MLHD in combination with genetic search algorithms delivers comparable results with algorithms using tree classifiers like RF. In this context, it is important to mention that the training data sets used for the evaluation of the linear discriminant coefficients were always based on small subsets of gene expression ratios (in chromosomes). In other words, the number of genes considered for LDA or MLHD classification (p = 5) was set to be smaller than the smallest group size (n = 6) technical replicates per time point) [39,47,48] which is a strong criterion to reduce overfitting [30].

The candidate genes identified with GA/MLHD are reliable because we were able to confirm the resulting list of the most discriminative features by two other optimization algorithms greedy as well as SA and by the tree classifier method random forest. We performed data preprocessing (including preselection) which is important for further analysis and took care of parameter optimization which is essential to avoid the tendency of overfitting in a multivariate approach.

### Prediction of gene-gene interactions

#### PCA

**Figure 2:**
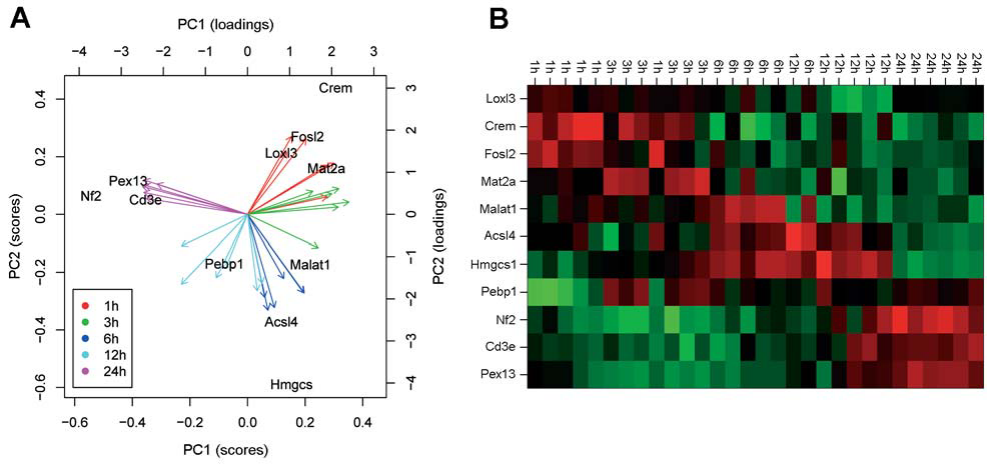
**Results of PCA analysis**. (A) Biplot of 11 candidate genes (scores) and time points after CRH treatment (loadings). The first two principal components (PC1 and PC2) were used to generate the biplot. In particular, correlations between the gene data points Pex13, Cd3e and Nf2 on the one hand as well as Crem and Fosl2 on the other hand are conspicuous. (B) The heat map represents the grouping of genes and time points by PCA. 24 h-replicates are completely separated whereas 1 h-and 3 h-replicates as well as 6 h-and 12 h replicates differentiate only partly based on the expression data. Positive and negative values of log_2 _expression ratios are colored in red and green, respectively. Black colored expression ratios illustrate no differential expression.

In order to test whether a separation of the expression profiles of the candidate genes into time points (after CRH treatment) can be achieved using unsupervised clustering methods, a principal component analysis (PCA) was performed. The underlying data matrix consisted of 11 rows for the genes and 30 columns for 5 time points including 6 technical replicates. The resulting scores and loadings from PCA for the objects (i.e. genes in terms of expression ratios) and variables (i.e. time points), respectively, were visualized by a biplot (Figure 2A) allowing for interpretation of relationships between them. Similar negative gene scores on the x-axis (PC1) of Pex13, Cd3e and Nf2, which are in the same region as the 24 h time point vectors, are well correlated with each other. Fosl2 and Crem, which show positive scores on PC1 and are located in the vicinity of the 1 h vectors, are also correlated. If the vectors point at the same region as the data points of the objects the gene expression is increased, otherwise it is decreased. Time points close to each other in Figure 2A have similar gene expression patterns. This is supported by the heatmap in Figure 2B, showing e.g. Fosl2 and Crem (with positive scores on the y-axis; PC2, Figure 2A) upregulated after 1 h and downregulated after 24 h of CRH treatment, whereas Pex13, Cd3e and Nf2 were downregulated after 1 h and upregulated after 24 h. Examples for poor correlation in their differential expression according to Figure 2A are Pex13 and Fosl2 as well as Nf2 and Acsl4 which have negative and positive scores on PC1, respectively. Furthermore, Figure 2A and 2B show that the expression patterns of the 11 candidate genes are able to clearly discriminate the time point groups 1 and 3 h, 6 and 12 h as well as the time point 24 h. Within the first 3 h CRH is known to regulate immediate early genes such as c-Fos [20]. Therefore, the PCA analysis reflects common transcriptional changes within the first 3 h of treatment including signaling molecules. Moreover, the correlation of the time points 6 and 12 h mirror similar processes on transcriptional level regulated by a late CRH response represented by genes involved in metabolic processes. Long-term effects of CRH were clearly discriminated at 24 h where the regulation of primary signaling molecules is no longer needed but specific downstream cellular processes are activated.

#### GeneNet

**Figure 3:**
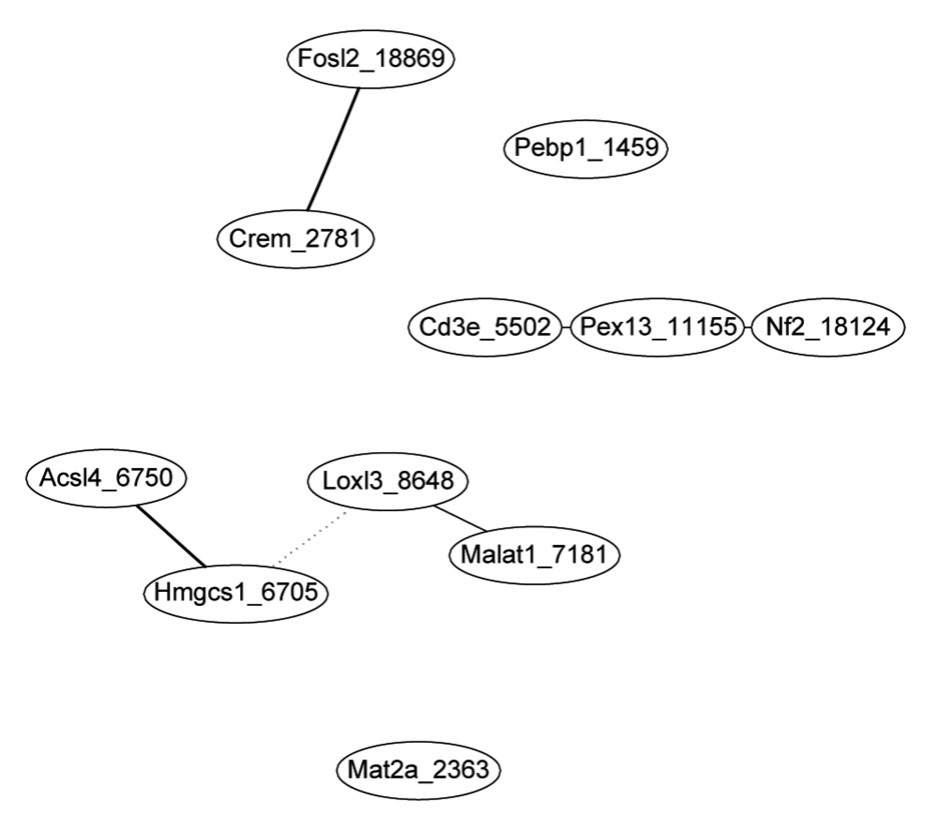
**Undirected graph computed by GeneNet for genes revealed by GALGO analyses**. GeneNet analysis was based on the expression ratios from the microarray of 11 genes. Solid lines in black depict positive partial correlation between genes. For the genes Pebp1 and Mat2a relations with other genes were not found by GeneNet whereas the dotted line between Hmgcs1 and Loxl3 represents a negative partial correlation.

The subset of 11 candidate genes with high frequency ranks derived from the supervised variable selection procedure and investigated by PCA was further analyzed by constructing a gene association network with help of the R package GeneNet [49], another unsupervised correlation method. Primarily, GeneNet was developed for analyzing gene expression (time series) data with focus on the interference of gene networks [31,50]. The resulting undirected graph from the GeneNet program is shown in Figure 3. We considered gene pairs with |*p_cor_*| > 0.35 and corresponding p-values < 0.05 at their edges and additionally unconnected nodes. The association network of putatively co-regulated genes consists of four main subnetworks with the gene clusters Fosl2-Crem, Cd3e-Pex13-Nf2, Acsl4-Hmgcs1 and Loxl3-Malat1 (Figure 3, where Hmgcs1 and Loxl3 are negatively partially correlated). These findings are in agreement with the above mentioned results of the PCA-an independent unsupervised clustering method-where most of the gene clusters were found to be correlated, in particular Fosl2-Crem, Pex13-Cd3e-Nf2 and Acsl4-Hmgcs1 (Figure 2A and 2B).

A complete discrimination (LOOCV = 1) into time points was achieved with sets of five genes (chromosomes) in the case of Pebp1, Mat2a, Crem, Hmgcs1 and Malat1 as well as in case of Mat2a, Crem, Cd3e, Fols2 and Malat1. The concordance of genes from these two chromosomes with genes in every subnetwork derived from GeneNet indicates that all four clusters including the two unconnected nodes (Figure 3) play an important role for the description of the overall time response.

#### Reconstruction of CRH signaling pathways by text mining

**Figure 4:**
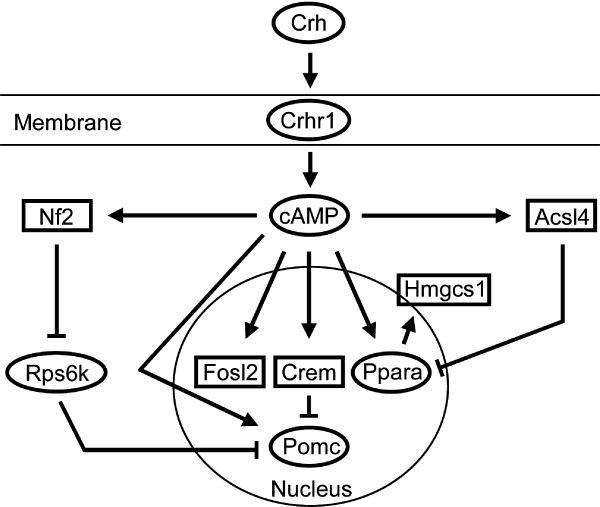
**Result of shortest path searches between all candidate genes investigated by GeneNet**. After manual curation of each interaction the resulting pathways were combined in this picture. Experimentally with qRT-PCR validated genes are drawn by rectangles and intermediates are indicated by circles. Lines with an arrowhead reflect positive regulation, other lines indicate inhibition.

To validate candidate interactions revealed in PCA and GeneNet analyses the literature mining software Pathway Studio was used. Direct and indirect protein-protein interactions, expression and promoter binding as well as regulation such as common regulators or targets were taken into account. Every connection found by Pathway Studio was confirmed manually and incorrectly associated interactions were excluded. No literature-based interaction was found for the GeneNet-built connection of Malat1 and Loxl3 (Figure 3), which is consistent with the weak correlation in the PCA results. In addition, for the negative partial correlation between Hmgcs1 and Loxl3 no relation was found using the Pathway Studio software, as was confirmed by PCA (Figure 2). Nf2, Pex13 and Cd3e clustered together in the GeneNet algorithm because of their regulation 24 hours following CRH stimulation. Therefore, these molecules should not be involved in acute signal transduction but in the modulation of CRH-dependent cellular processes such as proliferation or immune response [51-53]. Along these lines, all three genes have divergent functions. Nf2, a tumor suppressor, plays a critical role in cell proliferation by blocking growth factor receptor-dependent pathways [54]. Interestingly, a single nucleotide polymorphism in the Cd3e genes is associated with antidepressant treatment response [55]. The peroxisomal biogenesis factor Pex13 functions as protein transporter in peroxisomes and is related to fatty acid oxidation. The verification of the interactions predicted by GeneNet with the Pathway Studio software resulted in an indirect protein-protein interaction via SH3 domains. Pex13 contains an SH3 domain itself, whereas Nf2 and Cd3e can bind proteins, which in turn are capable of binding SH3 domains [56-59]. Moreover, a putative linkage of these three candidate genes to CRHR1-cAMP-mediated signal transduction was found for Nf2 (Figure 4). PKA phosphorylates Nf2, which triggers the dimerization with ezrin and causes cell growth [60]. As CRH is known to regulate cell proliferation Nf2 could be one of the responsible molecules mediating the CRH effect on cell growth [51,52]. Additionally, Nf2 is able to block MAP kinase signaling pathways [54] and thereby possibly affects CRHR1-regulated transcription.

The next group of interacting candidate genes containing Hmgcs1 and Acsl4 (Figure 4) is involved in lipid metabolism. Both genes were found to be connected via the peroxisomal proliferator-activated receptor α (PPARA) which maintains fatty acid homeostasis by induction of fatty acid oxidation and plays a role in controlling peroxisomal proliferation. Hmgcs1 was also found to be regulated on protein level after 100 nM CRH treatment of AtT-20 cells [19]. The Hmgcs1 gene contains a peroxisome proliferator response element (PPRE) in its promoter that binds PPARA/RXR heterodimers [61] and long-chain acyl-CoA synthetases (LC-ACS), like Acsl4, inhibit PPARA-mediated transcription [62]. Acsl4 transcription is activated by cAMP [63] and PPARA is phosphorylated by this second messenger [64] linking these genes with Gα_s _protein-coupled receptor signaling pathways.

The CRH/CRHR1-dependent regulation of genes involved in lipid metabolism strengthens a potential role of CRH as a modulator of metabolic function. Many psychiatric and neurological disorders share changes in metabolism [65,66]. Hmgcs1, e.g., together with other genes linked to fatty acid metabolism is upregulated by antipsychotics in human glioma cells [67].

The putatively connected candidate genes Crem and Fosl2 are both transcription factors. Crem is a modulator of cAMP responsive element (CRE)-dependent transcription [68] and is known to be regulated by CRH [20]. The expression of numerous genes such as CRH or tyrosine hydroxylase involved in psychiatric and neurodegenerative disorders, respectively, is regulated by Crem [69]. Fosl2 is a transcription factor of the Fos family, of which the immediate-early gene c-Fos is the most prominent member. Becquet and colleagues (2001) showed that upon CRH treatment transcription factors of the Fos-and Jun-family bind to the Pomc promoter and regulate its transcription [70]. Literature mining revealed cAMP as the connecting molecule for both transcription factors (Figure 4). cAMP-mediated induction of Crem leads to transcription of its inducible form Icer which is driven by an alternative intronic promoter [68]. In the case of Fosl2, cAMP leads to Fosl2-dependent transcriptional regulation of genes containing an AP1-binding site in their promoter [71,72]. As it is well known that CRH induces cAMP, and its downstream signaling cascades via a Gα_S _protein the GeneNet algorithm not only elucidated an interaction between Crem and Fosl2, but the result can be linked to CRHR1-dependent signaling pathways, especially since the Pomc promoter contains a cAMP-responsive element as well as an AP-1 binding site [73-75]. Crem and Fosl2 both showed an up-regulation within the first hour of CRH stimulation. Early regulated transcription factors Crem and Fosl2 may play an important role in the regulation of CRHR1-dependent signal transduction, probably by triggering or coordinating the transcription of secondary regulated genes.

Nf2, Acsl4, Crem and Fosl2 are known to be regulated by cAMP and thus targets of Gα_S_-protein-dependent signaling mechanisms. As CRH/CRHR1 promotes the synthesis of the second messenger cAMP the regulation of these four candidate genes by cAMP is likely to depend on direct CRH stimulation. As cAMP activates different signaling cascades via PKA such as CREB, L-type Ca^2+^channels and MAP kinase pathways, the distinct time-dependent differential regulation of candidate genes is likely to be stimulated by those different downstream pathways. Additionally, secondary effects of CRH-activated signaling such as expression and transactivation of transcription factors, e.g. of the AP1 family, lead to time-delayed changes in gene expression.

In summary, according to present knowledge, the inter-gene connections identified by the GeneNet algorithm were validated and additionally integrated into known CRHR1 signaling pathways.

#### Validation of CRH-regulated genes over time via qRT-PCR

To strengthen the biological relevance of the theoretical findings based on multivariate GALGO and unsupervised GeneNet algorithms the microarray data were partly confirmed by quantitative real-time PCR on all candidate genes that were analyzed by PCA and GeneNet, respectively, and subsequently verified by applying the Pathway Studio software. Total RNA isolated from two independent biological replicates at different time points was reverse transcribed and cDNA was analyzed in technical duplicates by qRT-PCR. As an internal standard the housekeeping genes Hprt and Gapdh were used. Both genes were not differentially regulated in AtT-20 cells by CRH stimulation.

**Figure 5:**
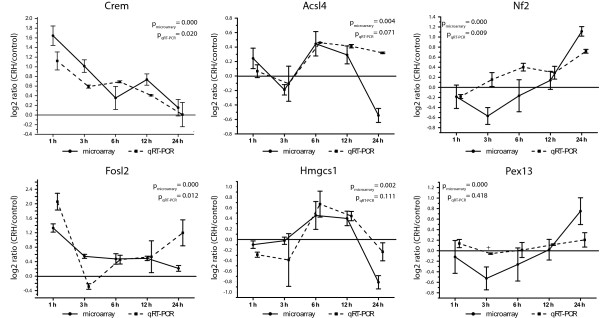
**qRT-PCR validation of the differential expression of six candidate genes at different time points**. Filled circles located on solid lines represent differential expression values from the microarray whereas filled squares on dashed lines show qRT-PCR expression values of AtT-20 cells independently treated with CRH related to their untreated controls and normalized to the house keeping gene Hprt. p-values were evaluated by ANOVA analyses.

We confirmed that after CRH stimulation the transcription of Crem was increased in the first 12 hours. The differential expression of Fosl2 was increased at the time points 1, 6, 12 and 24 h in the microarray as well as in the qRT-PCR. Acsl4 and Hmgcs1 showed increased mRNA levels in both analysis at 6 and 12 hours whereas Nf2 and Pex13 were upregulated by CRH after 12 and 24 hours (Figure 5). The expression level of Cd3e was out of the linear detection range of the Lightcycler instrument and thus not validated. With both internal references, Hprt and Gapdh, similar results were obtained. The microarray and qRT-PCR regulation values correlated well confirming the validity of the expression kinetics. Genes were defined as validated when the direction of regulation as determined by qRT-PCR was at least at one time point in agreement with the microarray result. To reveal the significance of the qRT-PCR in comparison to the microarray results ANOVA was performed. For Crem, Fosl2 and Nf2 the CRH-and time-dependent changes in expression are significant in microarray and qRT-PCR data and for Acsl4 the p-value shows a trend showing the reproducibility of the microarray results in independent material (Figure 5). For Hmgcs1 and Pex13 no significance in the ANOVA was obtained although the expression changes over time are similar in the qRT-PCR and in the microarray data. In the case of Hmgcs1 the variance between the samples is high, thus the analysis of more samples would help to get the results statistically significant. In the case of Pex13 the differential expression in the microarray analysis is very low and therefore difficult to validate with qRT-PCR although the regulation at 24 h was measured in both experiments.

The validation of the expression dynamics supported the clustering results of the GeneNet algorithm. Genes similarly regulated over time were considered as putative interaction partners. As the validated six candidate genes showed differential expression over time in an independent experiment, the co-regulated genes Crem and Fosl2, Acsl4 and Hmgcs1, Nf2 and Pex13 can be regarded as important mediators of CRH/CRHR1-dependent signaling pathway.

## Conclusions

To dissect CRHR1-dependent signaling pathways in detail, genome-wide expression profiling of CRH-stimulated AtT-20 cells was performed at five different time points. A combination of univariate preselection, multivariate discriminant analyses followed by unsupervised graphical models was employed to find genes significantly regulated by CRH/CRHR1-dependent mechanisms. Starting with more than 12000 expressed genes, we isolated a small subset of genes connected to CRHR1 signaling mechanisms. We focused on genes that occurred multiple times in GALGO analyses and contributed significantly to the discrimination of different time points following CRH treatment. Additional analyses using the state-of-the-art algorithm random forest as well as further optimization methods such as SANN and greedy, revealed similar results which strengthened the reliability of the GALGO results. Consequently, possible correlations between these genes were determined by PCA and GeneNet. Moreover, the differential expression of a subset of candidates was validated independently and determined interactions were confirmed via Pathway Studio software. This approach was able to condense the enormous dataset to a manageable subset of discriminative genes, which can now be subjected to detailed functional studies.

## Methods

### Cell culture

AtT-20 cells were obtained from the American Type Culture Collection (Manassas, VA) and cultured under standard conditions in Dulbecco's modified eagle medium (DMEM; Invitrogen, Karlsruhe, Germany) supplemented with 10% fetal calf serum (FCS; Invitrogen) and antibiotics (Invitrogen). The cells were maintained in a humidified 5% CO_2 _atmosphere at 37°C. After FCS deprivation for 18 hours cells in each experiment were treated with human/rat CRH (100 nM) (Bachem, Heidelberg, Germany) for 1, 3, 6, 12 and 24 h, respectively. In addition, untreated control cells were harvested at the same time points.

### RNA isolation and microarray hybridization

AtT-20 cells were harvested and total RNA was isolated with TRIzol^® ^reagent (Invitrogen) according to the manufacturer's protocol. RNA integrity was tested by gel electrophoresis. Amplified RNA (aRNA) synthesis and aRNA labeling were performed with the Amino Allyl MessageAmp™ aRNA Kit (Ambion, Austin, Texas) following the manufacturer's protocol. To exclude dye bias a dye-swap approach was chosen, i.e. one half of control AtT-20 aRNA and treated AtT-20 aRNA, respectively, was coupled to mono-reactive Cy3 and the other half to Cy5 N-hydroxysuccinimid (NHS) esters (Amersham). The labeled control and treated aRNA samples were mixed (Cy3-labeled control with Cy5-labeled treated samples and vice versa) and hybridized onto MPIP 24 k mouse cDNA arrays (Max Planck Institute of Psychiatry, Munich, Germany) [21]. In total, six technical replicates (three for each dye-coupling combination) were performed and scanned on a PerkinElmer Life Sciences ScanArray 4000 laser scanner (Rodgau-Jügesheim, Germany).

### Quantitative real-time PCR

The identity of selected candidate genes was verified by sequencing of the corresponding array clones (Sequiserve, Vaterstetten, Germany).

cDNA of independently treated AtT-20 cells was synthesized with SuperScript II-reverse transcriptase (Invitrogen) primed with oligo(dT) primers using 1 μg of total RNA according to the manufacturer's instructions. cDNA of untreated and 100 nM CRH-treated AtT-20 cells was analyzed by quantitative real-time PCR (qRT-PCR) using the LightCycler^® ^FastStart DNA MasterPLUS SYBR Green I reagent (Roche Diagnostics GmbH, Mannheim, Germany) according to manufacturer's instructions and different oligonucleotide primers (see Table 2). The experiments were performed in duplicates in the LightCycler^®^2.0 instrument (Roche Diagnostics, Mannheim, Germany) with the following PCR settings: initial denaturation at 95°C for 10 min; 40 cycles of denaturation (95°C for t_D _= 10 sec), annealing (T_A _= 56-65°C for t_A _= 4-5 sec) and elongation (72°C, t_E _= 7-13 sec). At the end of every run a melting curve (50-95°C with 0.1°C/sec) was measured to ensure the quality of PCR products. Crossing points (Cp) were calculated by the LightCycler^®^Software 4.0 (Roche Diagnostics, Mannheim, Germany) using the absolute quantification fit points method. Threshold and noise band were set manually in all compared runs at the same level. Relative gene expression was determined by the 2^-ΔΔCT ^method [76] using the real PCR efficiency calculated from an external standard curve, normalized to the housekeeping genes Hprt and Gapdh, respectively, and related to the data of untreated AtT-20 cells.

**Table 2:** Primers used for qRT-PCR validation

**gene**	**5' → 3'**
Acsl4 fwd	GGAGCCAAGCCAGAAAAC
Acsl4 rev	GCCTGTCATTCCAGCAATC
Crem fwd	ACATGCCAACTTACCAGATCC
Crem rev	TTTTCAAGCACAGCCACAC
Fosl2 fwd	GGTAGATATGCCTGGCTCGG
Fosl2 rev	TCATCTCTCCTTCTGCGGCC
Gapdh fwd	CCATCACCATCTTCCAGGAGCGAG
Gapdh rev	GATGGCATGGACTGTGGTCATGAG
Hprt fwd	ACCTCTCGAAGTGTTGGATACAGG
Hprt rev	CTTGCGCTCATCTTAGGCTTTG
Hmgcs1 fwd	AATGCCGTGAACTGGGTCG
Hmgcs1 rev	TGAGGTAGCACTGTATGGAGAGC
Nf2 fwd	TTCAAGAGATCACGCAACAC
Nf2 rev	TTCTCTCCTCCCACATTTCC
Pex13 fwd	TCCTGTTCTTTGCTGTTATCC
Pex13 rev	TCATCCTCACCACTTGCC

For validation we compared the expression ratios of six selected genes measured via qRT-PCR at each time point after CRH stimulation with the corresponding expression profiles from the microarray using statistical tests. One-way ANOVA on the log_2_-transformed expression ratios (treated versus control) with the factor time was applied using the *aov *function in the statistical software R utilizing partitioned error for replicates. Altogether four measurements of qRT-PCR normalized to Hprt, i.e. two biological and two technical replicates were considered in the ANOVA for each time point to determine statistical significance. In the case of the microarray data a two-way ANOVA with the factors time and dye-swap for six technical replicates was performed as described for the preselection process in preparation for supervised variable selection. In addition to the ANOVAs calculated separately for the qRT-PCR and microarray expression ratios the overall time response was considered if up-or down-regulation of the corresponding gene was measured equally in both analyses by visual inspection of the plots of the expression ratios against time points.

### Data normalization and preprocessing

Intensity extraction from the microarray scan images was accomplished by the fixed circle quantification method using QuantArray (PerkinElmer Life Sciences). The bottom 10% of the scan intensity values were defined as background and erased. Raw data were normalized using a lowess-based MA-smoother with print-tip correction implemented after Yang et al. [33] where M denotes the intensity ratio log_2_I(Cy3) -log_2_I(Cy5) or log_2_I(Cy5) -log_2_I(Cy3) and A the average intensity (log_2_I(Cy3) + log_2_I(Cy5))/2 or (log_2_I(Cy5) + log_2_I(Cy3))/2 of a spot signal. MA-plots are helpful to identify spot artifacts and to detect intensity-dependent patterns in the log ratios M. For the within-array normalization we used the robust scatter plot smoother *loess *(with the parameter *span *= 0.75) implemented in R statistical software [77] to perform a fit with a polynomial surface to the MA-plots of the raw data. The fitting procedure was done locally. The normalized intensity ratio were computed by the difference between the measured M-ratio and the predicted ratio from the loess regression. To reduce dye-biased effects on gene expression data a linear model (two-way ANOVA) considering the factors time and dye-swap was applied. The dye-swap is represented by the two levels [CRH-treated(Cy3)]/[control(Cy5)] and [CRH-treated(Cy5)]/[control(Cy3)]. A different effect of the dye combinations on the expression ratios of the six technical replicates will result in a significant interaction term of the ANOVA. Thus, by excluding gene expression ratios corresponding to a significant interaction between both factors the bias introduced by the different properties of the dyes was removed. Only spot signals which were detected at each point in time for a total of 30 two-colour arrays were taken into consideration. Factorial ANOVA was performed on log_2_-transformed data using the *lm *function in the statistical software R. The p-values from the ANOVA were adjusted for multiple testing using the false discovery rate (FDR) correction of Benjamini and Hochberg [78] implemented in the R-function *p.adjust*.

### Supervised variable selection

Before applying multivariate analysis a feature preselection was performed to eliminate not differentially expressed genes over time. We used a two-way linear model without interaction of the factors time and dye-swap. Genes were ranked according to their p-value after FDR-adjustment for multiple testing. To check if the expression ratios used in the two-way ANOVA are log_2_-normal distributed as well as if equal variances across groups exist, we performed Shapiro-Wilk's tests (with the R-function *shapiro.test*) and Levene's tests (using the R-function *levene.test*), respectively. In case of the Levene's test the factor variable *group *was set to five time points having six replicates each and the parameters *option *= trim.mean as well as *trim.alpha *= 0.25 were utilized.

A prerequisite for correct application of linear discriminant analysis (LDA) is the normality in the variables. Therefore, it was tested how many of the gene expression ratios used within the covariance matrix are log_2_-normal distributed (with the R-function *shapiro.test*). For verification which numbers of variables are suitable to avoid overfitting the classification error specifically the training error by resubstitution and test error by leave-one out cross-validation was plotted against the number of variables used in the LDA. Sets of variables (i.e. expression ratios) in the range of 2 to 30 were randomly drawn one thousand times each from the preselected data set and the averaged classification errors were calculated. Numbers of variables in the LDA should be avoided if the test error increases while the training error steadily decreases or don't change because then overfitting may have occurred.

### GA/MLHD

Genetic algorithm (GA) is a heuristic search procedure based on natural selection according to the following stages (for details see [26,79]):

1. Creation of chromosomes that are subsets of variables

2. A fitness function is used to evaluate the ability to predict the group membership of each sample for each chromosome

3. Selection of chromosomes with a fitness higher than a predefined value and stop of the procedure otherwise continuation with stage 4

4. Reproduction of chromosomes relatively to its fitness; crossover between two randomly selected parent chromosomes; random insertion of mutations (new genes) in chromosomes; repetition from stage 2 until an accurate chromosome is determined.

GALGO, an R package based on genetic algorithm search procedure was applied for supervised multivariate variable selection [25]. Starting point of the GA is the random creation of chromosomes with a size of five features (stage 1). For the fitness function the leave-one out cross-validation (LOOCV) procedure in combination with the classifier from discriminant analysis was chosen to evaluate the fitness value, which is defined as the classification accuracy of a selected chromosome. The fitness function controls chromosome selection in the genetic algorithm (stage 2). We utilized the maximum likelihood (MLHD) method for classification which is equivalent to linear discriminant analysis (LDA) [26,79]. To minimize overfitting the maximum likelihood method implemented in GALGO was restricted to subsets of five variables (chromosome size).

In addition, a gene ranking was based on the frequency of each feature in chromosomes satisfying the goal fitness (stage 3). The search parameters in the genetic algorithm included up to 2500 iterations (maximum solutions) to collect a large number of variable combinations and an estimated classification accuracy with optimizing criteria of 100% (goal fitness). The maximum number of generations (stage 4) was set to 200 because thousands of generations would end up in overfitting. Finally, the topmost 50 genes were selected. In order to validate gene rank stability and to isolate genes occurring in several analyses, the feature selection algorithm was repeated separately four times with the same input data and parameter settings.

### Greedy algorithm

We applied the *greedy.wilks *function from the klaR package in R to the preselected microarray dataset and used LOOCV in conjunction with LDA to obtain the most frequently identified genes for setting up a predictive model. Based on a small part of the data the greedy algorithm iteratively adds one locally optimal component after another to extend the data structure until an optimal solution is reached. The *greedy.wilks *method performs a stepwise forward variable selection using the Wilk's Lambda criterion [80]. Starting point is the choice of the gene with the lowest p-value from the overall F-Statistic.

Leaving out one sample (technical replicate) at a time, we applied the *greedy.wilks *method to select genes showing p-values from F-statistic of the partial Wilk's Lambda (*p.value.diff*) smaller than a predefined significance level (*niveau *= 0.001). Partial Wilk's Lambda is defined by the difference between two statistical models in which the one model contains the new variable while the other does not. A LDA classifier was trained with the selected gene expression ratios and the group membership was predicted for the excluded test dataset. By repeating the described procedure for all samples and counting the number of times a gene was picked out, we generated a ranked list with the most frequently identified genes and evaluated the classification error.

### Simulated annealing

We developed an algorithm for input selection of subsets of variables (INSEL) which uses simulated annealing (SA), LDA as well as LOOCV, and produces a ranked list of variables (genes) with high discriminative power. In our input selection algorithm we implemented the *optim *function with *method *= SANN of the stats package in R. SANN is a variant of simulated annealing given by Belisle [81] and uses the Metropolis function for acceptance of probability. The method SANN requires an initial set of variables (*sq*) to be optimized over, a target function to be minimized (*fn*) and a function that generates a new candidate combination (*gr*) as well as control parameters such as the maximum number of iterations (*maxit*) and the starting temperature for the cooling schedule (*temp*). In our case *sq *represents the complete preselected gene set from the microarray by random selection of a small subset of genes (*chromosome size *= 5). The fitness function *fn *evaluates the classification error using LDA and LOOCV based on the gene expression ratios in the subset whereas the *gr *function introduces a mutation in the subset. A mutation is defined as an exchange of one gene in a chromosome against a randomly selected new gene from the dataset. The control parameter *maxit *gives the total number of function evaluations and was set to 1000. Following Press et al. [82] we calculated the mean variation of the classification error (representing a generalized energy) over 10000 randomly selected chromosomes from the dataset in order to evaluate the starting temperature *temp*. The temperature acts as a control parameter for the search area and is gradually lowered until no further improvement of the fitness function is detected.

We applied the described procedure by collecting up to 2500 solution chromosomes. Based on all solutions we filtered the list for chromosomes that show an estimated classification accuracy of 100% and ranked the genes according to their frequency. Finally, the topmost 50 genes were selected. Altogether four runs were performed with the same parameter settings.

### Random forest

We applied the R-function *randomForest *according to Breiman [83] to the whole preselected microarray data using standard parameter settings, i.e. *ntree *= 500, *mtry *= $\sqrt{number\;of\;genes}$, and *nodesize *= 1. The arguments *ntree*, *mtry *and *nodesize *are defined by the number of trees, the number of input variables tried at each split and the minimum size of the terminal nodes, respectively. It was reported that the default value for *mtry *is often a good choice [84]. To produce a ranked list of the variables (genes) according to the mean decrease in Gini criterion the parameter *importance *was set to TRUE. The analysis described was repeated several times in order to examine the stability of the results.

### Modelling of gene-gene interactions

#### PCA

To reveal groups of genes that act together we first used an unsupervised method-principal component analysis-to describe the correlation structure of the selected genes. Correlations between genes were derived from PCA of gene expression patterns using the *prcomp *function with default settings in the R package stats. The gene (row) by time point (column) matrix of expression ratios was analyzed by PCA to determine the scores for the objects (gene expression ratios) and the loadings for the variables (time points). After mean centering of the gene expression ratios the ratios as well as time points were simultaneously plotted with help of a biplot [85,86]. The PCR was applied on a small subset of candidate genes derived from the multivariate analysis by GA/MLHD.

#### GeneNet

Additionally, a search for correlations, which cannot be explained by other variables, was performed. These partial correlations are used as a measure of conditional independence and are the basis for graphical Gaussian models (GGMs). For construction of a gene association network-GGMs that represent multivariate dependencies-the R package GeneNet (version 1.1.0) [49] was used. GeneNet contains functions for calculating shrinkage estimators (*ggm.estimate.pcor*) and for assigning statistical significance for the edges in a network (*ggm.test.edges*). The undirected graph for a small subset of genes from the feature selection procedure with GA/MLHD was calculated with the parameter *method *= dynamic in the function *ggm.estimate.pcor *by foregoing mean centering of the expression ratios.

#### Text mining

To verify the predicted network from GeneNet the corresponding gene-gene interactions were searched for in all PubMed abstracts with the help of a text mining program (Pathway Studio 5.0, Ariadne Genomics) based on the Natural Language Processing (NLP) Technology.

## Abbreviations

ANOVA: analysis of variance; FDR: false discovery rate; CRH: corticotropin-releasing hormone; CRHR: corticotropin-releasing hormone receptor; ERK 1/2: extracellular signal-regulated kinase; GA: genetic algorithm; GGMs: graphical Gaussian models; HPA: hypothalamic-pituitary-adrenal; KNN: K-nearest neighbour; LDA: linear discriminant analysis; MAPK: mitogen-activated protein kinase; MLHD: maximum likelihood; NC: nearest centroid; PCA: principal component analysis; PKA: protein kinase A; POMC: proopiomelanocortin; qRT-PCR: quantitative real-time polymerase chain reaction: SA: simulated annealing; SVM: support vector machines.

## Authors' contributions

DT designed and performed the bioinformatic data analyses including univariate preselection, multivariate GALGO analysis as well as principal component and GeneNet analyses followed by literature mining. CG designed and carried out the biological experiments including cell culture treatments, RNA preparation, microarray hybridization and scanning and qRT-PCR analyses. DT and CG wrote the manuscript. BP performed the normalization of the raw microarray data. CK, MP and PW assisted in the microarray performance and analyses. FH and WW directed the work. GW wrote the manuscript and together with JD supervised the project and revised the manuscript. All authors read and approved the final manuscript.

## Supplementary Material

Additional file 1 **MA plots**. MA-plots of the spot signals from 48 pins of the raw and normalized microarray data including loess fit curves Click here for file

Additional file 2 **SupplementaryTable 1**. Genes regulated by CRH in murine corticotrope AtT-20 cells as identified by GALGO analyses Click here for file

Additional file 3 **Generalization curves**. Training (resubstitution) and test (leave-one out cross-validation) error as a function of the number of variables used in the LDA Click here for file

Additional file 4 **R code**. Implemented R code of the variable selection algorithm INSEL based on simulated annealing and LDA (For download of the R software: http://cran.r-project.org/). Click here for file
