# Supplementary figures and images for: Deducing corticotropin-releasing hormone receptor type 1 signaling networks from gene expression data by usage of genetic algorithms and graphical Gaussian models
Source: BMC Syst Biol. 2010 Nov 19;4:159. doi: 10.1186/1752-0509-4-159 (PMC3002901; doi:10.1186/1752-0509-4-159)

12 h - raw data

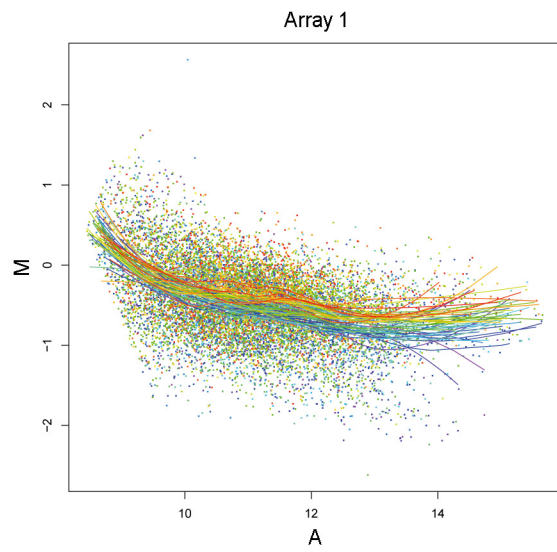

12 h - normalized data

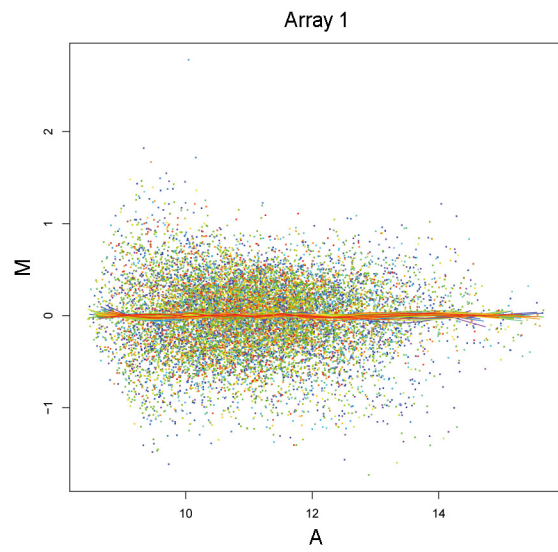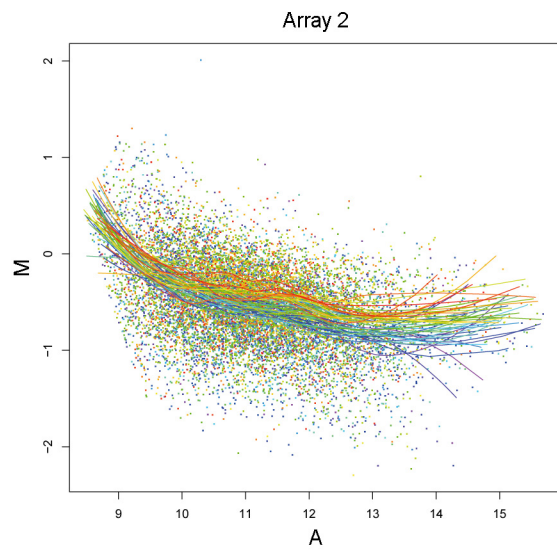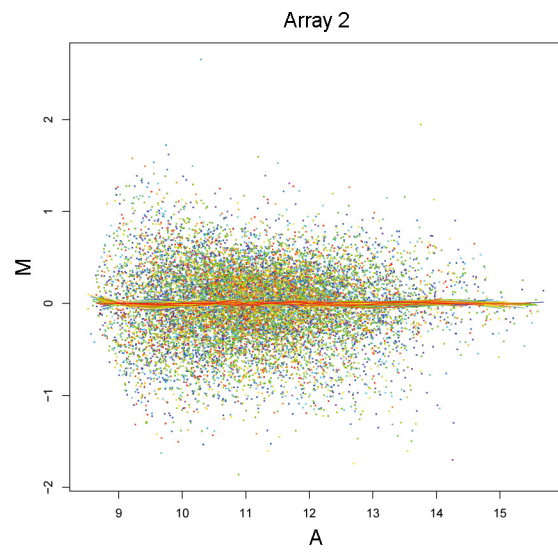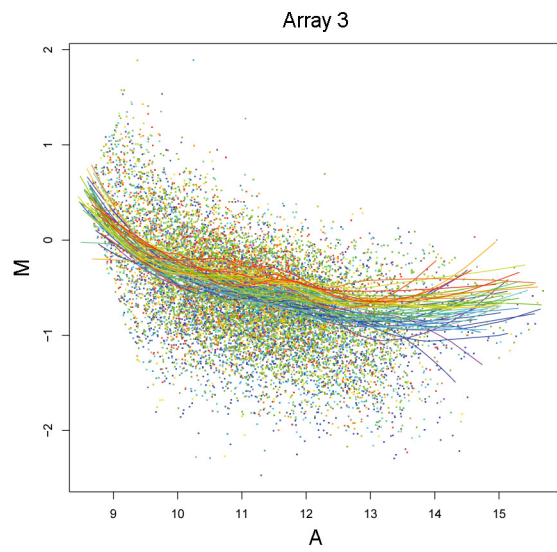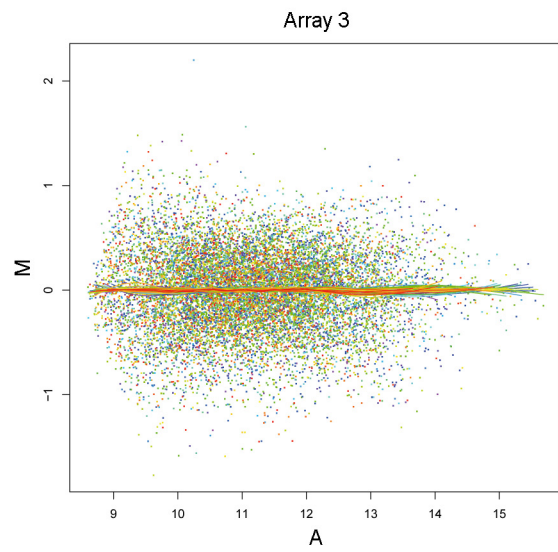

12 h - raw data

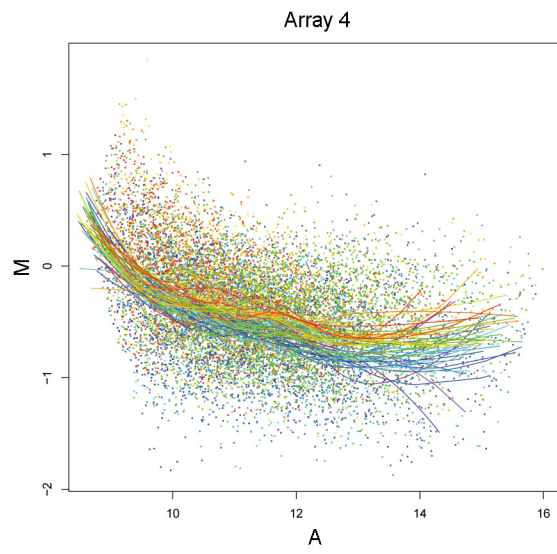

12 h - normalized data

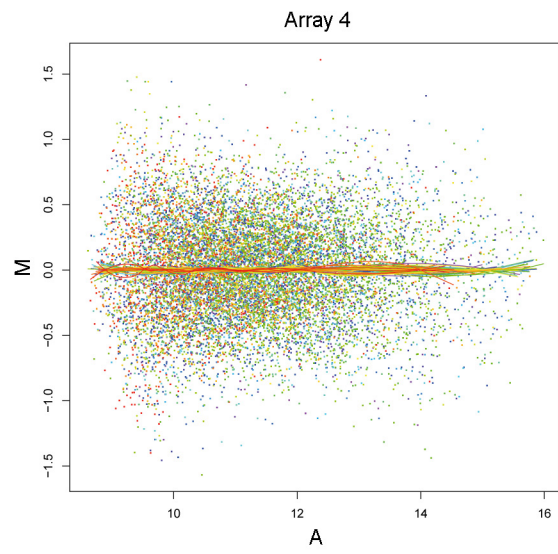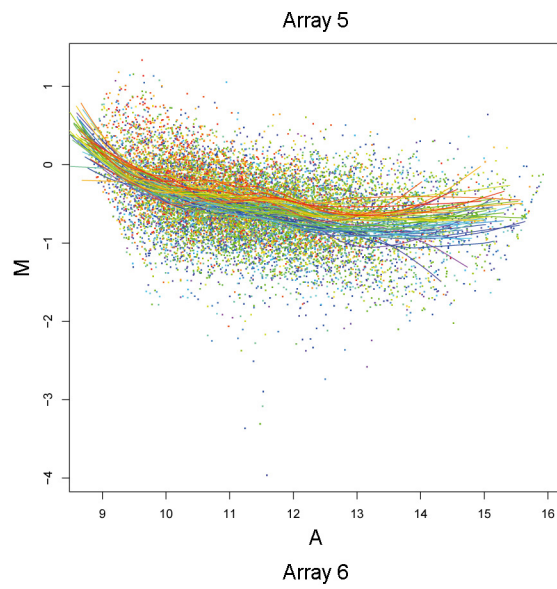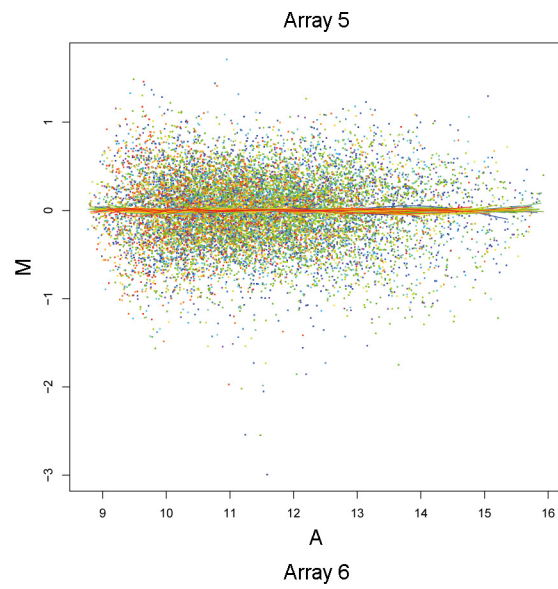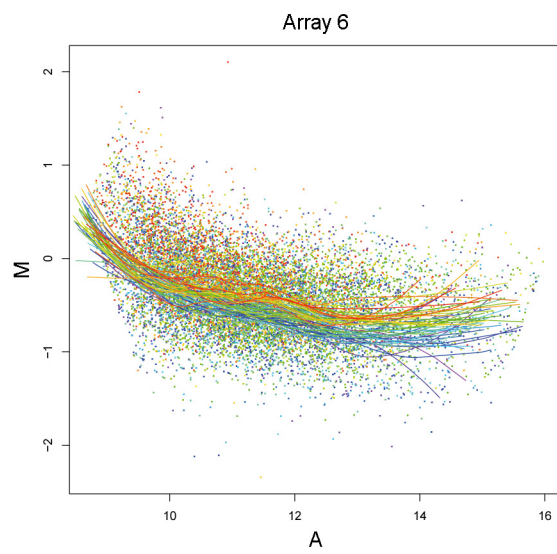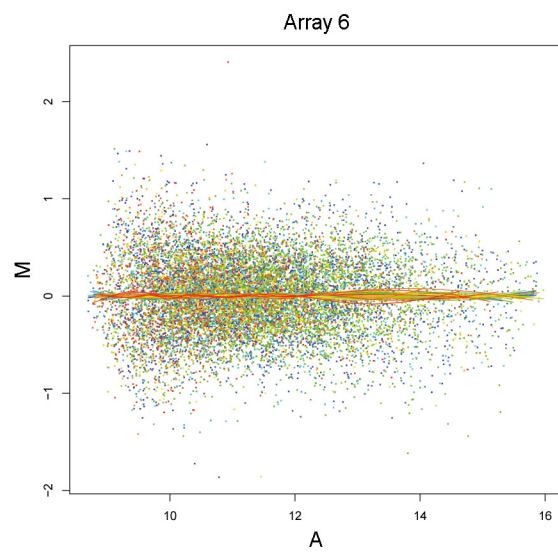

Supplement: Additional file 1 — MA plots. MA-plots of the spot signals from 48 pins of the raw and normalized microarray data including loess fit curves [file 1752-0509-4-159-S1.PDF]

Classification error

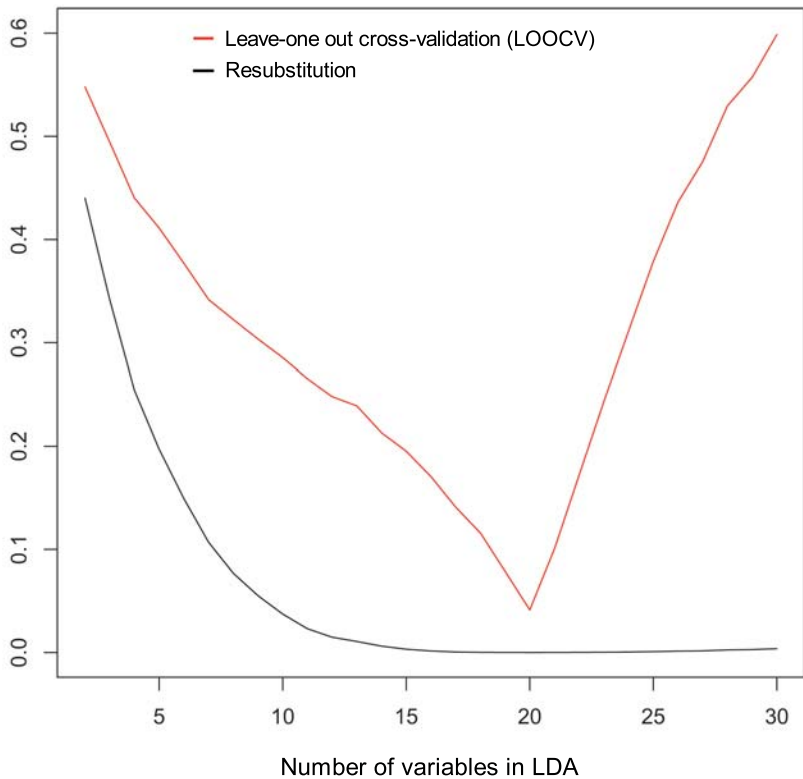

Supplement: Additional file 3 — Generalization curves. Training (resubstitution) and test (leave-one out cross-validation) error as a function of the number of variables used in the LDA [file 1752-0509-4-159-S3.PDF]
