# Supplementary material for: Deducing corticotropin-releasing hormone receptor type 1 signaling networks from gene expression data by usage of genetic algorithms and graphical Gaussian models
Source: BMC Syst Biol. 2010 Nov 19;4:159. doi: 10.1186/1752-0509-4-159 (PMC3002901; doi:10.1186/1752-0509-4-159)
Supplement: Additional file 2 — SupplementaryTable 1. Genes regulated by CRH in murine corticotrope AtT-20 cells as identified by GALGO analyses [file 1752-0509-4-159-S2.PDF]

Supplementary Table 1

Genes regulated by CRH in murine corticotrope AtT-20 cells as identified by GALGO analyses[a]

Spot ID	Accession Number	Gene	Description	Gene Rank Analysis 1	Analysis 2	Analysis 3	Analysis 4
Transcription							
**4700**	**AA254318**	**4930522L14Rik**	**RIKEN cDNA 4930522L14 gene**	**7**	**1**	**2**	**31**
**2781**	**AI846359**	**Crem[b]**	**CAMP responsive element modulator**	**2**	**20**	**20**	**16**
**18869**	**BG072398**	**Fosl2[b]**	**Fos-like antigen 2**	**39**	**9**	**17**	**21**
15702	AI415706	Zfp213	Zinc finger protein 213	111	27	14	20
6620	AI835157	Neurod1	Neurogenic differentiation 1	50	81	68	26
20962	AI451115	Tcof1	Treacher Collins Franceschetti syndrome 1, homolog	67	13	90	73
7196	AI854843	Gli3	GLI-Kruppel family member GLI3	117	99	195	39
5075	AI854209	Sf3a1	Splicing factor 3a, subunit 1	268	165	28	54
19524	AI851247	Fosl2	Fos-like antigen 2	40	189	75	227
2656	AI848408	Id2	Inhibitor of DNA binding 2	142	164	205	24
322	AI839114	Sqstm1	Sequestosome 1	239	25	155	265
20114	AA140515	Ythdc1	YTH domain containing 1	166	40	380	161
Nucleotide binding							
**1459**	**CD776350**	**Pebp1**	**Pebp1 phosphatidylethanolamine-binding protein 1**	**18**	**46**	**21**	**9**
1550	AA270909	Top2a	Topoisomerase (DNA) II alpha	53	17	98	61
5931	AI843104	Ddx3x	DEAD/H (Asp-Glu-Ala-Asp/His) box polypeptide 3, X-linked	78	65	118	47
7671	AI838523	Scaf1	SR-related CTD-associated factor 1	27	113	112	59
8060	AA389327	Skiv2l2	Superkiller viralicidic activity 2-like 2 (S. cerevisiae)	137	145	46	44
21313	AA734490	Abcd4	ATP-binding cassette, sub-family D (ALD), member 4	28	307	256	67
Signal transduction							
**5502**	**AA185709**	**Cd3e**	**CD3 antigen, epsilon polypeptide**	**4**	**32**	**8**	**15**
5926	AI450767	Mapk1	Mitogen activated protein kinase 1	108	70	135	46
11281	AI449143	Calm1	Calmodulin 1	36	106	104	189
Receptor activity							
2383	AI847223	Sema6c	Sema domain, transmembrane domain (TM), and cytoplasmic domain, (semaphorin) 6C	203	208	56	37
Transport							
**11155**	**AI450121**	**Pex13[b]**	**Peroxisomal biogenesis factor 13**	**22**	**12**	**7**	**18**
5887	AI841677	Tmed3	Transmembrane emp24 domain containing 3	42	52	23	102
3233	AI117694	Snx11	Sorting nexin 11	26	30	226	22
1296	AI605736	Slc6a11	Solute carrier family 6 (neurotransmitter transporter, GABA), member 11	60	140	30	95
12864	AI850801	Tmed7	Transmembrane emp24 protein transport domain containing 7	86	214	33	40
8536	AI847502	Dld	Dihydrolipoamide dehydrogenase	150	44	173	25
23109	AI843695	Slc37a3	Solute carrier family 37 (glycerol-3-phosphate trans-porter), member 3	202	168	97	49
17859	AI842864	Rabif	RAB interacting factor	45	101	166	224
15400	AI839164	Tmed10	Transmembrane emp24-like trafficking protein 10 (yeast)	130	47	270	118
21723	AI846151	Rnuxa	RNA U, small nuclear RNA export adaptor	330	48	188	83
Protein binding							
3496	AI847845	Pcdha4	Protocadherin alpha 7	44	160	10	62
4999	AI893639	Anapc1	Anaphase promoting complex subunit 1	144	37	110	100
22642	AI450905	Cep164	Centrosomal protein 164	220	237	11	36
3139	AI847065	Nktr	Natural killer tumor recognition sequence	187	39	79	239
Protein biosynthesis							
18265	AA048130	Rps27l	Ribosomal protein S27-like	62	29	4	30
24169	AA549039	Taf4b	TAF4B RNA polymerase II, TATA box binding protein (TBP)-associated	29	103	91	124
20471	AA182979	Eif2c3	Eukaryotic translation initiation factor 2C, 3	218	305	202	42
Negative regulation of protein kinase activity							
**18124**	**AI323564**	**Nf2[b]**	**Neurofibromatosis 2**	**10**	**22**	**25**	**45**

Table S1 *Continued*

Spot ID	Accession Number	Gene	Description	Gene Rank Analysis 1	Analysis 2	Analysis 3	Analysis 4
Negative regulation of protein kinase activity							
1333	AI842320	Nf2	Neurofibromatosis 2	68	180	55	43
Hydrolase activity							
19583	AI844881	Ddx3x	DEAD/H (Asp-Glu-Ala-Asp/His) box polypeptide 3, X-linked	24	73	153	23
15972	AI849987	Smpdl3a	Sphingomyelin phosphodiesterase, acid-like 3A	30	111	95	65
23726	AA268030	Dpysl5	Dihydropyrimidinase-like 5	77	107	39	94
15156	W41539	Pnpla1	Patatin-like phospholipase domain containing 1	213	19	82	285
18134	AI848680	Nt5dc2	5'-nucleotidase domain containing 2	37	157	251	158
Transferase activity							
**2363**	**AI849149**	**Mat2a**	**Methionine adenosyltransferase II, alpha**	**5**	**36**	**16**	**3**
15752	AI426672	Dnttip2	Deoxynucleotidyltransferase, terminal, interacting protein 2	123	162	36	35
16573	AI447731	Cmas	Cytidine monophospho-N-acetylneuraminic acid synthetase	34	8	217	175
21246	W35091	Coq2	Coenzyme Q2 homolog, prenyltransferase (yeast)	17	204	203	137
Oxidoreductase activity							
**2336**	**AI451727**	**0610012D14Rik**	**RIKEN cDNA 0610012D14 gene**	**35**	**45**	**27**	**34**
4730	AI661450	Idh2	Isocitrate dehydrogenase 2 (NADP+), mitochondrial	32	85	117	273
19778	AA259765	Prdx5	Peroxiredoxin 5	76	35	341	108
Lipid metabolism							
**6750**	**AI846046**	**Acsl4**[b]	**Acyl-CoA synthetase long-chain family member 4**	**38**	**18**	**1**	**33**
**16977**	**AI843116**	**Hmgcs1**	**3-hydroxy-3-methylglutaryl-Coenzyme A synthase 1**	**33**	**24**	**273**	**10**
**6705**	**AI841574**	**Hmgcs1**[b]	**3-hydroxy-3-methylglutaryl-Coenzyme A synthase 1**	**3**	**3**	**5**	**346**
6715	AA060369	Hmgcr	3-hydroxy-3-methylglutaryl-Coenzyme A reductase	61	193	47	127
Apoptosis							
4606	AI841410	Tax1bp1	Tax1 (human T-cell leukemia virus type I) binding protein 1	23	97	76	125
12328	AI327173	Aplp1	Amyloid beta (A4) precursor-like protein 1	210	127	161	41
15853	AA615635	Egln3	EGL nine homolog 3 (C. elegans)	368	195	50	364
Cytoskeleton							
9477	AI835922	Kif3a	Kinesin family member 3A	65	14	37	60
12686	AI429298	Kif3a	Kinesin family member 3A	19	38	62	72
21425	NM_008744	Ntn1	Netrin 1	8	11	178	14
8837	AA200002	Kif17	Kinesin family member 17	118	42	142	145
21350	AI854113	Sdc2	Syndecan 2	235	28	41	291
6645	AI846131	Kif3c	RIKEN cDNA 1110002L01 gene	13	314	262	345
Mitochondrion							
4880	AI840657	Cox7a2l	Cytochrome c oxidase subunit VIIa polypeptide 2-like	75	55	26	4
9331	AI839512	Timm17a	Translocator of inner mitochondrial membrane 17a	173	77	32	38
10489	AI847956	Pigy	Phosphatidylinositol glycan anchor biosynthesis, class Y	121	49	175	70
Biological process unknown							
13207	AA718111	Fgfr1op	Fgfr1 oncogene partner	11	34	24	51
15947	AI841252	Cnpy2	Canopy 2 homolog (zebrafish)	214	95	35	119
6037	AI847392	Gbas	Glioblastoma amplified sequence	158	43	45	243
Others							
**7181**	**AI227072**	**Malat1**	**Metastasis-associated lung adenocarcinoma transcript 1 (non-coding RNA)**	**1**	**2**	**3**	**1**
**19276**	**AA816221**	**6720467C03Rik**	**RIKEN cDNA 6720467C03 gene**	**12**	**5**	**6**	**13**
**2698**	**AI846018**		**Transcribed locus**	**15**	**7**	**19**	**12**
**8648**	**AA269410**	**Loxl3**	**Lysyl oxidase-like 3**	**9**	**16**	**12**	**17**
Others							
**3931**	**AI450057**	**A430097D04Rik**	**DNA segment, Chr 15, Brigham & Women's Genetics 0759 expressed**	**25**	**26**	**31**	**19**
19692	W58944	3200002M19Rik	RIKEN cDNA 3200002M19 gene	20	23	67	6
15482	AI465375	Misc12	MIS12 homolog (yeast)	16	21	74	11
3185	AI853444		Predicted gene, ENSMUSG00000063277	57	31	13	27
5478	AA387159	Wdr40c	WD repeat domain 40C	6	105	84	8

Table S1. *Continued*

Spot ID	Accession Number	Gene	Description	Gene Rank Analysis 1	Analysis 2	Analysis 3	Analysis 4
Others							
22725	AI447476	Mfap3	Microfibrillar-associated protein 3	46	72	109	74
16322	AI661037	Neil3	Nei like 3 (E. coli)	43	53	9	198
20310	AI448474		Transcribed locus	51	4	222	29
1103	AI849707	Myeov2	Myeloma overexpressed 2	104	150	54	7
16577	AI837598			64	188	38	28
14534	AI413090	BC037034	CDNA sequence BC037034	176	33	18	106
9017	AI837674	2610040E16Rik	Leucine rich repeat containing 40	41	87	48	167
5666	AA259694	Suv420h2	Suppressor of variegation 4-20 homolog 2 (Drosophila)	101	62	183	2
6201	AI837840			63	143	29	126
8023	AI323897	Tmod2	Tropomodulin 2	244	6	66	128
5491	AI847942	Sept4	Septin 4	66	172	194	32
17312	AI845689	Lamp1	Lysosomal membrane glycoprotein 1	182	116	42	134
3000	AA415398	MGC73851	Expressed sequence AA415398	14	58	133	270
9279	AA437817	Tfpi	Tissue factor pathway inhibitor	48	126	160	147
4267	AI449074		Transcribed locus	47	108	64	272
14161	AI841985		Transcribed locus	141	50	93	218
6684	AI842055	AW554918	Expressed sequence AW554918	136	15	72	305
5427	AI842990		Transcribed locus	107	286	134	5
4831	AI894247	Tubb5	Tubulin, beta 5	205	152	44	141
1382	AA120647			49	228	163	130
11316	AA433626	Thnsl2	Threonine synthase-like 2 (bacterial)	249	166	40	129
20965	AI839696	Fkbp2	FK506 binding protein 2	93	41	255	231
20153	AI853067	Csnk1a1	Casein kinase 1, alpha 1	87	134	43	368
8047	AI449795	Usp48	Ubiquitin-specific peptidase 48	245	75	350	48
13118	AI854682	BC005561	CDNA sequence BC005561	21	300	49	358
22422	AI414820	Rrp1b	Ribosomal RNA processing 1 homolog B (S. cerevisiae)	112	10	387	234
5299	AI839515	Tbcd	Tubulin-specific chaperone d	31	223	345	165
7845	AI850268		Transcribed locus	342	200	34	211
7590	AI842091	Tgoln1	Trans-golgi network protein	273	199	330	50
1852	AI450720		Transcribed locus	289	342	22	205

[a]Functional classification was based on Gene Ontology (GO) categories according to NCBI. All genes were obtained by four separated GALGO analyses of the complete list of preselected genes and merged in this table. Those genes with boldface occur in four analyses among the topmost 50 ranked genes or occurred twice in at least three runs and were – after elimination of RIKEN clones and unknown transcripts – chosen for modeling of interactions with GeneNet. Genes that fall in the same GO category are sorted in ascending order by the sum of the Gene Ranks for all four analyses.

[b]Candidate genes validated over time by qRT-PCR.
